# Supplementary material for: Arousal state transitions occlude sensory-evoked neurovascular coupling in neonatal mice
Source: Commun Biol. 2023 Jul 17;6:738. doi: 10.1038/s42003-023-05121-5 (PMC10352318; doi:10.1038/s42003-023-05121-5)
Supplement: Supplementary file 2 — Supplementary Information [file 42003_2023_5121_MOESM2_ESM.pdf]

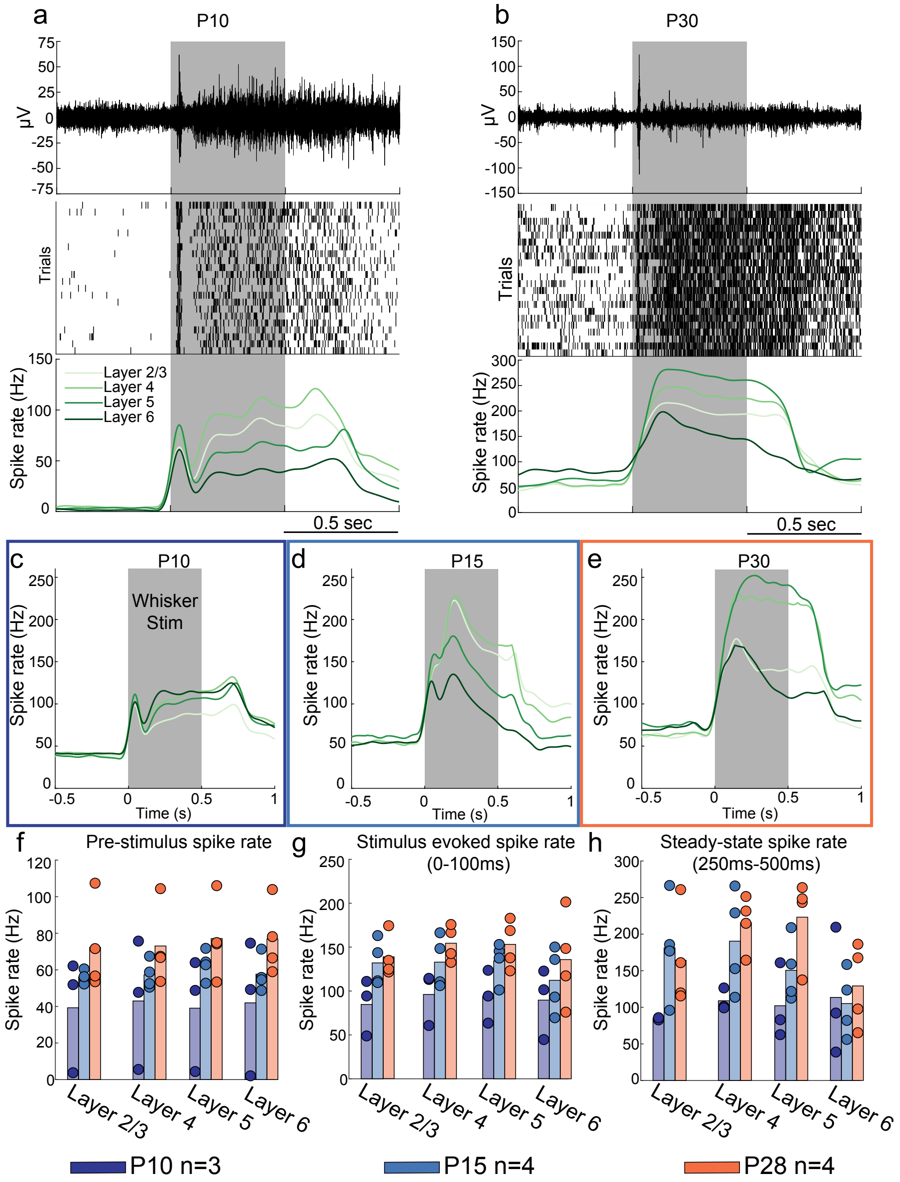

Supplementary Figure 1. Sensory-evoked spiking in the neonatal mouse. a. Neural responses to whisker stimulation in a P10 mouse. Top, whisker stimulus (grey shaded region), increases multi-unit activity for duration of stimulus in P10 animals. Middle, spike raster from an L5 neuron showing whisker stimulation reliably evokes spiking. Bottom: PSTH showing stimulation increases activity across all cortical layers. b. Neural responses to whisker stimulation in a P30 mouse. Layout is the same as in b. Note higher background rate. c-e. Top. Average PSTH for P10, P15, and P30 mice. Bottom, Spike rates P10, n=3 mice, P15 and 30 n=4 mice each. f. Pre-stimulation baseline spike rate by layer and age. Bars denote means, circles individual animals. g. Same as f, but for the early stimulus evoked response h. Same as g except for steady-state stimulus-evoked response.

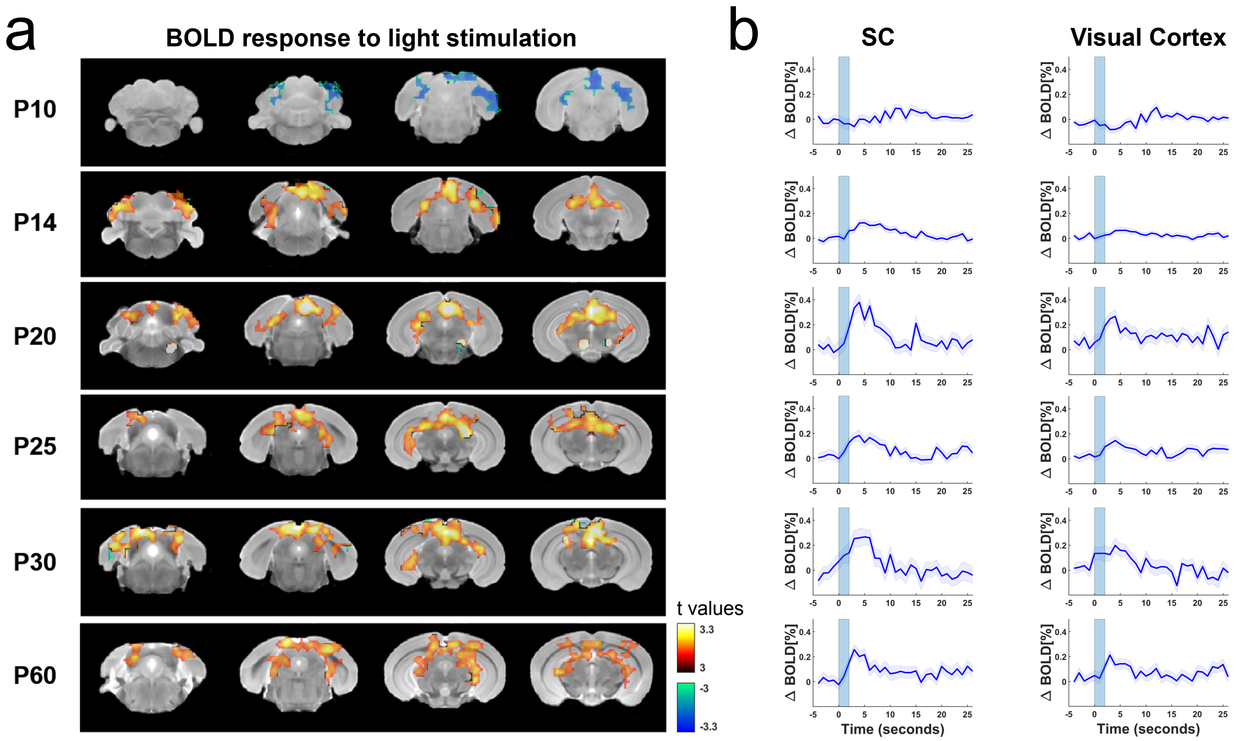

Supplementary Figure 2. Light stimulation evoked BOLD response in neonate mice. a. BOLD activity in response to visual stimulation at different postnatal stages. Only brain slices including the superior colliculus or primary visual cortex are shown. Pseudo colored voxels demonstrate positive (red) or negative (blue) BOLD response that is significantly different from baseline (two sample t-test, linear mixed model, $p < 0.05$, FDR corrected, N = 18,27,19,21,17,20 for age P10, P14, P20, P25, P30, P60, respectively). The BOLD responses in the superior colliculus and primary visual cortex are negative at P10, turn positive on P15, and gradually becomes adult-like from P20 to P60. b. Averaged time courses of BOLD signals across activated voxels in the superior colliculus and primary visual cortex, respectively. Blue shaded bars indicate the on time of light stimulation (duration = 2 sec).

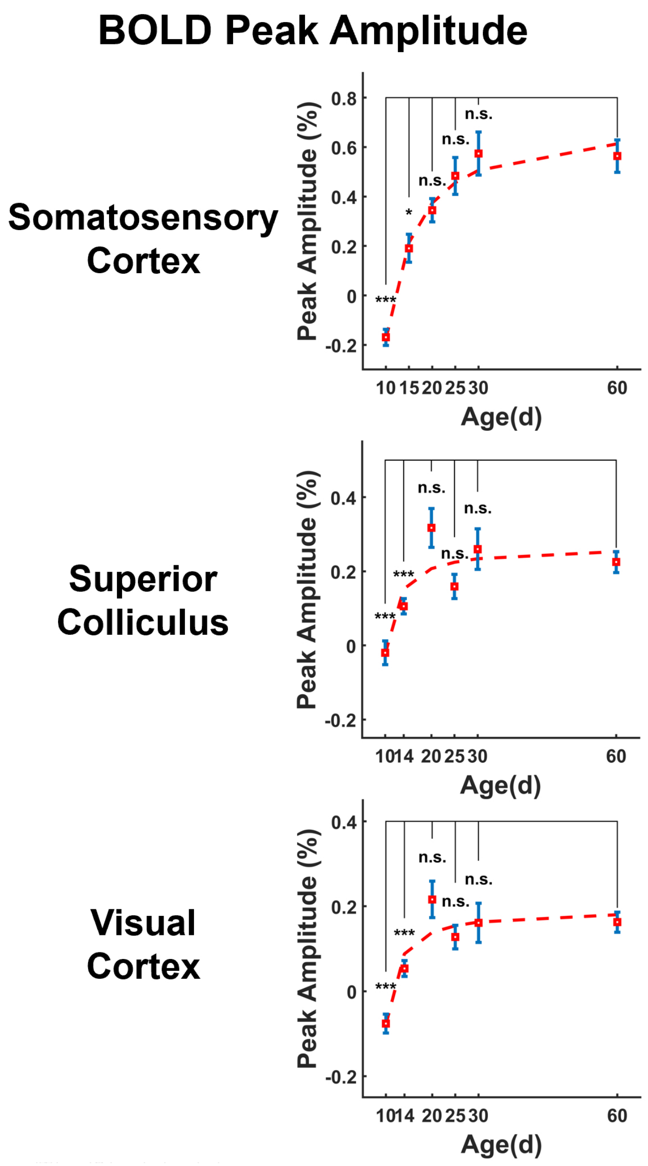

Supplementary Figure 3. BOLD peak amplitude across different age groups. For each epoch, the BOLD time course was first averaged across all activated voxels in each region of interest (ROI). The peak amplitude for the epoch was determined as the maximal BOLD response within 8 sec after the start of the stimulation. Error bar indicates the standard error of the mean, green dots represent individual trials.. A trend curve is fitted to determine the peak amplitude change during development.

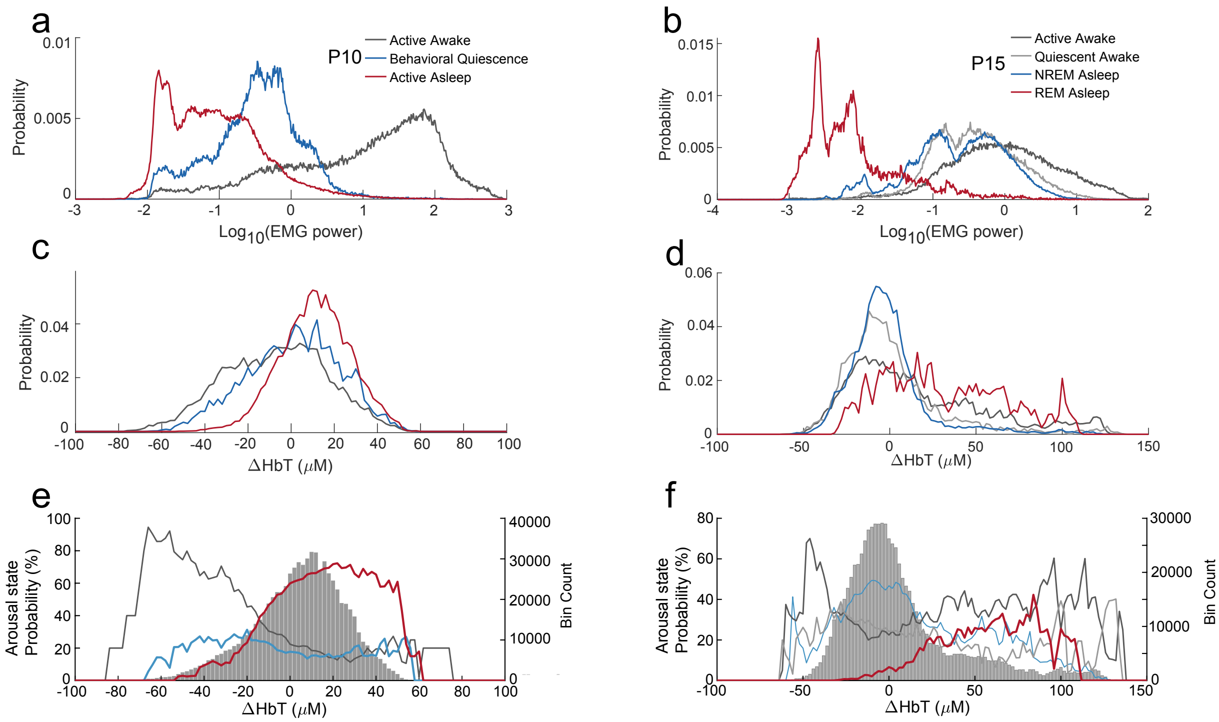

Supplementary Figure 4. Distribution on EMG power and Δ[HbT] by arousal state. a,b. Nuchal EMG power distribution for the different arousal states in P10 and P15 mice. Active/REM sleep consists of the lowest muscle tone, while active awake had the highest. c,d. Normalized distribution of Δ[HbT] for each arousal state. Note the rightward shift of REM/active asleep Δ[HbT] values. e,f. Probability of arousal state based on Δ[HbT]. Due to occurrence of vasodilation during locomotion, and relative rarity of REM events, large increases in blood volume are not indicative of arousal state on their own, and need to be paired with additional measure such as EMG to determine arousal state.

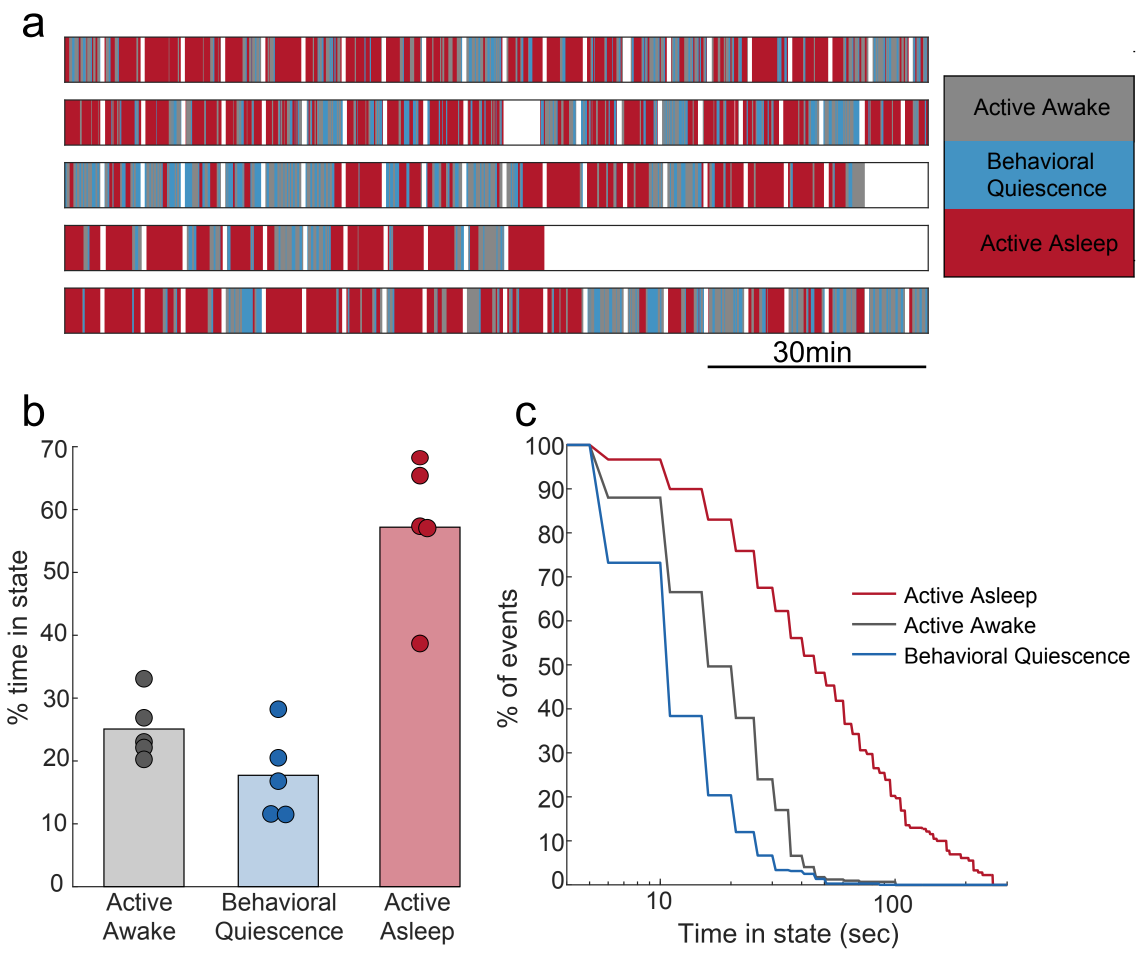

Supplementary Figure 5. P10 animals spend the majority of imaging session in active sleep and rapidly transition between wake and sleep states. a. Hypnograms of during two hours of imaging for five P10 mice at five second resoution White bins denote gaps in recordings. b. P10 animals spend over half of their time in active sleep during imaging experiments. c. Survivor plots of event duration by event type. Active sleep events are the most frequent in number and longest in duration, while periods of awake quiescence are brief.

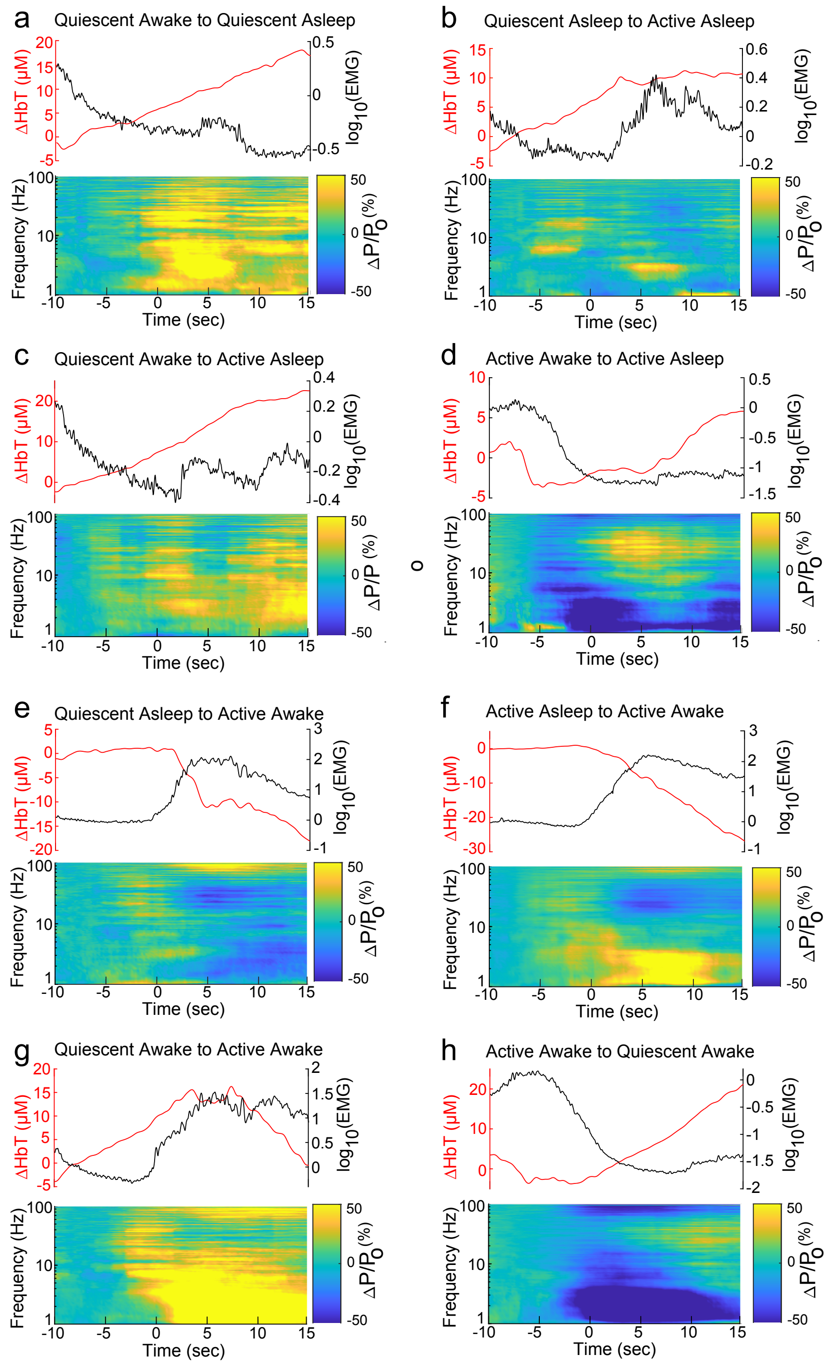

Supplementary Figure 6. Arousal states drive large changes in the EMG, hippocampal LFP, andΔ [HbT] in P10 mice. a-h Peri-transition EMG, LFP, and Δ[HbT] are plotted relative to the arousal state transition.

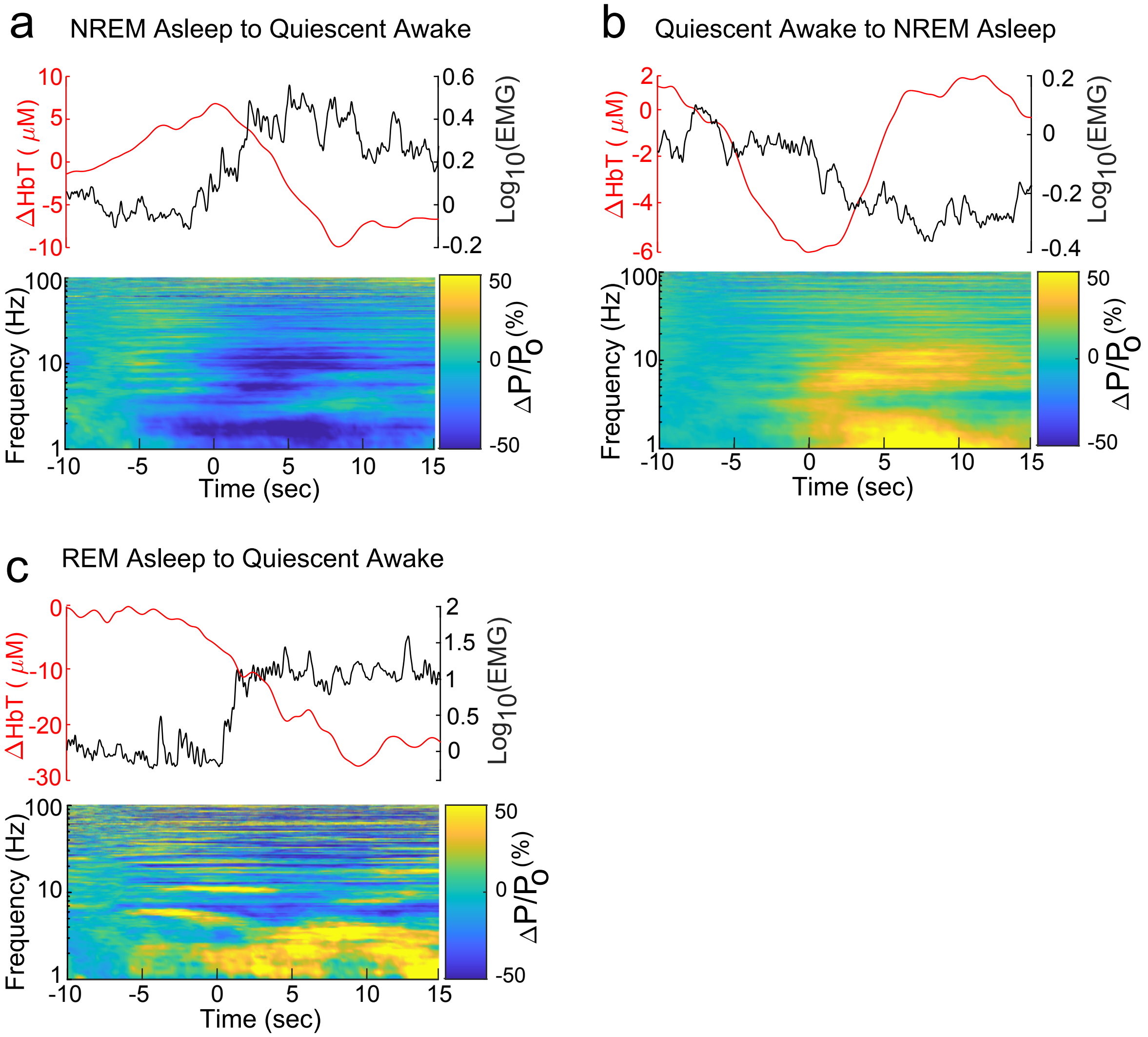

Supplementary Figure 7. Additional arousal state transitions for P15 mice. a-c. Peri-transition EMG, hippocampal LFP, and Δ[HbT] are plotted relative to the arousal state.
