# Supplementary material for: Arousal state transitions occlude sensory-evoked neurovascular coupling in neonatal mice
Source: Commun Biol. 2023 Jul 17;6:738. doi: 10.1038/s42003-023-05121-5 (PMC10352318; doi:10.1038/s42003-023-05121-5)
Supplement: Supplementary file 3 — Description of Additional Supplementary Files [file 42003_2023_5121_MOESM3_ESM.pdf]

Description of Additional Supplementary Files

File name: Supplementary Data 1

Description: Source data behind Figures 1-6.
