# Supplementary material for: Arousal state transitions occlude sensory-evoked neurovascular coupling in neonatal mice
Source: Commun Biol. 2023 Jul 17;6:738. doi: 10.1038/s42003-023-05121-5 (PMC10352318; doi:10.1038/s42003-023-05121-5)
Supplement: Supplementary file 5 — Reporting Summary [file 42003_2023_5121_MOESM5_ESM.pdf]

nature portfolio

Corresponding author(s): Patrick Drew

Last updated by author(s): Jun 14, 2023

Reporting Summary

Nature Portfolio wishes to improve the reproducibility of the work that we publish. This form provides structure for consistency and transparency in reporting. For further information on Nature Portfolio policies, see our Editorial Policies and the Editorial Policy Checklist.

Statistics

For all statistical analyses, confirm that the following items are present in the figure legend, table legend, main text, or Methods section.

n/a	Confirmed	
☐	☒	The exact sample size (*n*) for each experimental group/condition, given as a discrete number and unit of measurement
☐	☒	A statement on whether measurements were taken from distinct samples or whether the same sample was measured repeatedly
☐	☒	The statistical test(s) used AND whether they are one- or two-sided<br>*Only common tests should be described solely by name; describe more complex techniques in the Methods section.*
☐	☒	A description of all covariates tested
☐	☒	A description of any assumptions or corrections, such as tests of normality and adjustment for multiple comparisons
☐	☒	A full description of the statistical parameters including central tendency (e.g. means) or other basic estimates (e.g. regression coefficient) AND variation (e.g. standard deviation) or associated estimates of uncertainty (e.g. confidence intervals)
☐	☒	For null hypothesis testing, the test statistic (e.g. *F*, *t*, *r*) with confidence intervals, effect sizes, degrees of freedom and *P* value noted<br>*Give P values as exact values whenever suitable.*
☒	☐	For Bayesian analysis, information on the choice of priors and Markov chain Monte Carlo settings
☐	☒	For hierarchical and complex designs, identification of the appropriate level for tests and full reporting of outcomes
☐	☒	Estimates of effect sizes (e.g. Cohen's *d*, Pearson's *r*), indicating how they were calculated

Our web collection on statistics for biologists contains articles on many of the points above.

Software and code

Policy information about availability of computer code

Data collection	Optical imaging data was acquired using custom code: https://github.com/DrewLab/LabVIEW-DAQ<br>Multielectrode data was acquired using Neuronexus acquisition software
Data analysis	Optical imaging and multielectrode data was analyzed in Matlab.

For manuscripts utilizing custom algorithms or software that are central to the research but not yet described in published literature, software must be made available to editors and reviewers. We strongly encourage code deposition in a community repository (e.g. GitHub). See the Nature Portfolio guidelines for submitting code & software for further information.

Data

Policy information about availability of data

All manuscripts must include a data availability statement. This statement should provide the following information, where applicable:

- Accession codes, unique identifiers, or web links for publicly available datasets
- A description of any restrictions on data availability
- For clinical datasets or third party data, please ensure that the statement adheres to our policy

Data will be deposited on Dryad upon acceptance. DOI:10.5061/dryad.k6djh9wbt

Research involving human participants, their data, or biological material

Policy information about studies with human participants or human data. See also policy information about sex, gender (identity/presentation), and sexual orientation and race, ethnicity and racism.

Reporting on sex and gender	n/a
Reporting on race, ethnicity, or other socially relevant groupings	n/a
Population characteristics	n/a
Recruitment	n/a
Ethics oversight	n/a

Note that full information on the approval of the study protocol must also be provided in the manuscript.

Field-specific reporting

Please select the one below that is the best fit for your research. If you are not sure, read the appropriate sections before making your selection.

☒ Life sciences ☐ Behavioural & social sciences ☐ Ecological, evolutionary & environmental sciences

For a reference copy of the document with all sections, see nature.com/documents/nr-reporting-summary-flat.pdf

Life sciences study design

All studies must disclose on these points even when the disclosure is negative.

Sample size	Sample size was chosen to be consistent with previous studies in the literature (e.g. Coelho-Santos et al., PNAS, 2019)
Data exclusions	none
Replication	Sensory stimulation was performed multiple times per animal, as described in text.
Randomization	No randomization was performed
Blinding	No experimental treatments we compared, and no blinding was performed.

Reporting for specific materials, systems and methods

We require information from authors about some types of materials, experimental systems and methods used in many studies. Here, indicate whether each material, system or method listed is relevant to your study. If you are not sure if a list item applies to your research, read the appropriate section before selecting a response.

Materials & experimental systems

n/a	Involved in the study	
☒	☐	Antibodies
☒	☐	Eukaryotic cell lines
☒	☐	Palaeontology and archaeology
☐	☒	Animals and other organisms
☒	☐	Clinical data
☒	☐	Dual use research of concern
☒	☐	Plants

Methods

n/a	Involved in the study	
☒	☐	ChIP-seq
☒	☐	Flow cytometry
☐	☒	MRI-based neuroimaging

Animals and other research organisms

Policy information about studies involving animals; ARRIVE guidelines recommended for reporting animal research, and Sex and Gender in Research

Laboratory animals	Swiss Webster mice, both males and females were used, ages P8-adult. Ages are reported in figures.
Wild animals	n/a
Reporting on sex	Both
Field-collected samples	n/a
Ethics oversight	Experiments were approved by the Penn State Institutional Animal Care and Use Committee (IACUC)

Note that full information on the approval of the study protocol must also be provided in the manuscript.

Magnetic resonance imaging

Experimental design

Design type	task-based block design
Design specifications	There were 10 blocks in each scan, and each block is 30 seconds.
Behavioral performance measures	n/a

Acquisition

Imaging type(s)	functional MRI
Field strength	7T
Sequence & imaging parameters	Gradient echo EPI, FOV = 1.6 × 1.6 cm2, matrix size = 64x64, slice thickness = 0.75 mm, TE = 15ms. TR = 1s, flip angle = 60 degrees.
Area of acquisition	whole brain scan

Diffusion MRI ☐ Used ☒ Not used

Preprocessing

Preprocessing software	Matlab R2019a. The brain region was extracted by brain mask. Data were subjected to temporal linear detrending and spatial smoothing with a Gaussian kernel size of 0.5 mm.
Normalization	Imaging data of different age groups were aligned to structural templates of the corresponding age through rigid body registration. The imaging data was not normalized to template since the images aligned well with the age-specific templates.
Normalization template	The templates were downloaded from Center of Magnetic Resonance Microimaging at John Hopkins Medical Institute. Images were not normalized.
Noise and artifact removal	motion parameters were regressed
Volume censoring	Volumes with FD larger than 0.125 mm (half of the inplane voxel size) were discarded together with their immediate adjacent volumes using the Matlab software. Scans with more than 80% volumes removed were discarded.

Statistical modeling & inference

Model type and settings	linear mixed-effect model (within subject variability modeled as the random effect) was to estimate the group-level data
Effect(s) tested	Coherence to the stimulation frequency was tested. ANOVA or factorial design were not used.

Specify type of analysis: ☐ Whole brain ☐ ROI-based ☒ Both

Anatomical location(s)	The anatomical templates were nonlinearly registered to the Allen Mouse Brain Atlas by the ANTs software to determine anatomical ROI locations.

Statistic type for inference<br>(See Eklund et al. 2016)	Cluster-wise. Cluster size equal or larger than 10 voxels defined by two-dimensional eight-connected neighborhood
Correction	FWE-corrected threshold of P = 0.05

Models & analysis

n/a	Involved in the study
☒	☐ Functional and/or effective connectivity
☒	☐ Graph analysis
☒	☐ Multivariate modeling or predictive analysis
